# Phenolic Compounds in Nectar of *Crataegus monogyna* Jacq. and *Prunus spinosa* L.

**DOI:** 10.3390/plants14132064

**Published:** 2025-07-06

**Authors:** Katja Malovrh, Blanka Ravnjak, Mitja Križman, Jože Bavcon

**Affiliations:** 1 Biotechnical Faculty, University of Ljubljana, University Botanic Gardens Ljubljana, Ižanska 15, 1000 Ljubljana, Slovenia; katja.malovrh@bf.uni-lj.si (K.M.); blanka.ravnjak@bf.uni-lj.si (B.R.); 2 Department of Analytical Chemistry, National Institute of Chemistry, Hajdrihova 19, 1001 Ljubljana, Slovenia

**Keywords:** blackthorn, hawthorn, nectar, phenolic compounds

## Abstract

*Crataegus monogyna* Jacq. and *Prunus spinosa* L. are common spring-flowering species in Slovenia. They occur in large stands and sometimes overgrow in unmanaged meadows. They are known as an excellent source of nectar for bees and other pollinators. Phenolic compounds are known as antioxidant for both pollinators and plants. We were interested in comparing plant species in terms of their phenolic compound content: rutin, quercetin, (iso)quercitrin, chlorogenic acid, and hyperoside. Their nectar was obtained from both plant species in 2024 in Ljubljana and the area surrounding Ljubljana. We took 96 samples of each species. The nectar was sampled with microcapillary tubes and analysed by HPLC. When studying the influence of abiotic factors on the concentration of phenolic compounds, the correlations were weak, so we cannot say with certainty which environmental factors affect which phenolic compounds and in what way. Rutin is predominant in the nectar of *P. spinosa* and chlorogenic acid is predominant in the nectar of *C. monogyna*. Hyperoside is found in the lowest concentration in both plant species. We found that although *C. monogyna* secreted much less nectar at midday, it was more concentrated in phenolic compounds at this time than in the morning. In *P. spinosa*, nectar secretion was highest in the morning, and the concentration of phenolic compounds was also highest in the morning.

## 1. Introduction

Plants provide food to pollinators in the form of nectar and pollen, and pollinators transfer the pollen, which adheres to their bodies, to the pistil of the flower, thus pollinating the flower. Nectar is primarily intended to attract pollinators and is a source of nutrition for them [1,2,3]. However, other roles of nectar have been discovered in recent years. Pyke [4] and Nepi et al. [5] found that plants use nectar to manipulate insects to their advantage; sometimes they even attract insects to use their faeces.

Nectar contains 30–90% water and up to 70% sugars, nitrogen compounds, organic acids, pigments, essential oils, vitamins, and minerals [6,7]. Nicolson and Thornburg [6] also mention lipids, antioxidants, phenols, and terpenoids. Nectar also contains other compounds, mainly alkaloids, non-protein amino acids and other compounds that can make nectar toxic or even repel certain pollinators [8].

Plants produce a range of secondary metabolites that have a variety of functions, mainly defence against herbivores and microbes, but that can also attract pollinators [9,10]. Secondary plant metabolites also include phenols or phenolic compounds, which are also found in nectar. The nectar of a single plant species can contain several dozen different phenolic compounds. Nectar and honey both contain concentrations of phenolic compounds. The same phenolic compounds found in nectar can also be found in honey. The concentration of secondary metabolites in nectar is lower than in pollen. Some are thought to act by deterring animals that do not pollinate flowers [11]. These substances are thought to enter the nectar via the plant’s own vasculartissue, similar to sugars [8]. Secondary metabolites may include alkaloids, phenolic glycosides, and other phenolic compounds [9].

The presence of phenolic compounds is also thought to affect bee colonies. Rutin is thought to be the most common phenolic compound in nectar [12]. It is also important because it has a protective effect against certain insecticides (protecting their neurosystem), which then do not harm pollinators due to its presence. Rutin even protects honeybees against the parasitic mite *Varroa* sp., which is a major problem in Slovenia and causes neurological problems in honeybees [13]. Quercetin is one of the most common flavonoids in plants [14] and is also preferred by bees. Furthermore, (iso)quercitrin and, in particular, quercetin can affect honeybee colonies by affecting their hormones, which can induce the colony to rear more queens [15,16]. Although chlorogenic acid is considered a common phenolic compound in nectar [12,17,18], it is thought to be toxic to bees and high levels can deter bees from pastures [8,19]. Hyperoside is secreted by plants as a defence mechanism when they are exposed to stress factors such as too high or too low temperatures or too much UVB radiation. It has antioxidant effects and increases cell viability [20].

Based on our observations over many years, woody species of the Rosaceae family in particular have been shown to be more honey-producing [21]. The concept of honey flow is a characteristic of plant species that defines the plant as a source of bee forage. Nectar, pollen, and honeydew are all sources of bee forage [22,23]. Factors that have a major influence on nectar quality and quantity are air humidity and temperature [24], day length [25], evaporation [6,26], and soil nutrient content [27].

Previous research on Rosaceae nectar has shown that glucose and fructose are the most abundant sugars [28,29,30] and that they also vary between plant taxa [31]. Each plant species has a specific type of nectar. Nectar composition and concentration can also vary within species, between populations in a given area, or even between individual plants at the flower level [32,33,34]. Environmental factors also influence composition in different populations [35]. In view of all this, we were interested to know whether *P. spinosa* and *C. monogyna* also have the same content of phenolic compounds and whether these concentrations vary according to environmental factors. We were interested in the content of five specific phenolic compounds—rutin, hyperoside, chlorogenic acid, quercetin, and (iso)quercitrin—in two woody plant species, namely *P. spinosa* and *C. monogyna*.

## 2. Results

### 2.1. Quantity of Nectar in the Plant Species Studied

**Figure 1:**
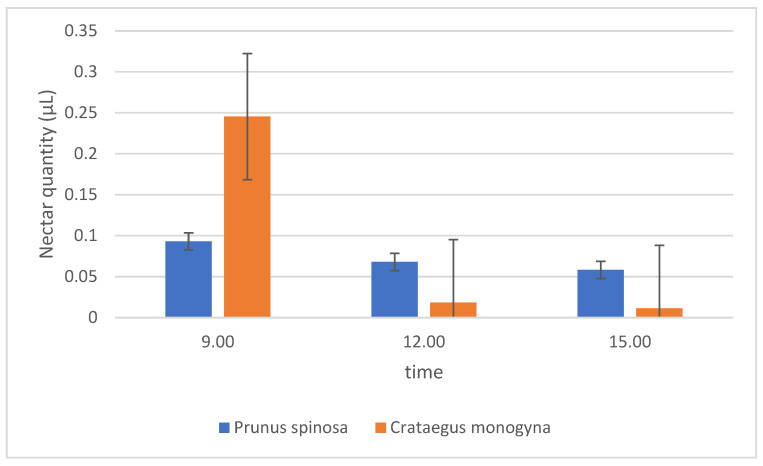
Average nectar amount secreted at three different times of the day. In one column, there are 32 samples for each hour of each species (N = 96).

*C. monogyna* produced between 0.01 and 0.25 μL of nectar per flower per day, while *P. spinosa* produced 0.06–0.09 μL (Figure 1). *C. monogyna* produced 0.09 μL and *P. spinosa* produced 0.07 μL, and, in the morning, *C. monogyna* (0.25 μL) had much higher nectar secretion than *P. spinosa* (0.09 μL). *P. spinosa* had about the same nectar production throughout the day, with a slight decrease towards the end of the day, but *C. monogyna* had much higher production in the morning than at midday and in the afternoon. The sampling of nectar on this species was often not possible in the afternoon due to the small amounts of nectar produced at that time of the day.

### 2.2. Content of Phenolic Compounds Studied in the Nectar of P. spinosa and C. monogyna

Comparison of the average phenolic compound concentrations (Table 1) showed that *C. monogyna* had a higher total phenolic compound concentration (61 μg/mL). *P. spinosa* had a lower total concentration of phenolic compounds (54 μg/mL), but much more rutin, while other phenolic compounds were less abundant. In *C. monogyna*, the predominant compound was chlorogenic acid, while hyperoside was the least abundant. *P. spinosa* was also very low in hyperoside but had rutin as the main compound.

### 2.3. Effect of Environmental Factors on Phenolic Compounds

#### 2.3.1. Effect of Environmental Factors on Phenolic Compounds in *P. spinosa* Nectar

Using Spearman’s correlation coefficient (Table 2), we found weak positive correlations between rutin and air temperature, soil temperature, and soil moisture, indicating that as these three abiotic factors increased, so did the rutin concentration. The correlation between (iso)quercitrin and soil temperature was weak and negative, meaning that as soil temperature increased, the concentration of (iso)quercitrin decreased. No other correlations were found.

#### 2.3.2. Effect of Environmental Factors on Phenolic Compounds in *C. monogyna* Nectar

The correlation between soil and air temperature and the concentration of chlorogenic acid, rutin, (iso)quercitrin, and hyperoside was negative and weak (Table 3), meaning that as the value of abiotic factors increased, the concentration of phenolic compounds decreased. Absolute humidity and UVB were negatively and weakly correlated with chlorogenic acid and rutin concentrations, and UVB was negatively and weakly correlated with (iso)quercitrin. All of these environmental factors have an impact on the lower concentrations of the phenolic compounds listed above. The correlation between soil temperature and the concentration of chlorogenic acid, rutin, (iso)quercitrin, and hyperoside was weak and negative, indicating that the concentration of these phenolic compounds decreased with increasing soil temperature. The correlation between air temperature and the concentration of rutin, (iso)quercitrin, and hyperoside was weak and negative, indicating that the concentration of these phenolic compounds decreased with increasing air temperature. However, the correlation between air temperature and chlorogenic acid was moderate and negative, indicating that there was a stronger correlation between air temperature and chlorogenic acid than other phenolic compounds and that increasing air temperature had a decreasing effect on chlorogenic acid concentrations. The correlation between absolute humidity and chlorogenic acid and rutin was weak and negative, indicating that the concentration of these two phenolic compounds decreased with increasing absolute humidity. The correlation between UVB and chlorogenic acid, rutin, and (iso)quercitrin was weak and negative, indicating that increasing UVB led to lower concentrations of these three phenolic compounds in the nectar. There was no correlation between soil humidity and phenolic compounds in *C. monogyna* nectar.

### 2.4. Effect of the Hour (Time of Day) on Phenolic Compounds

#### 2.4.1. Prunus Spinosa

**Figure 2:**
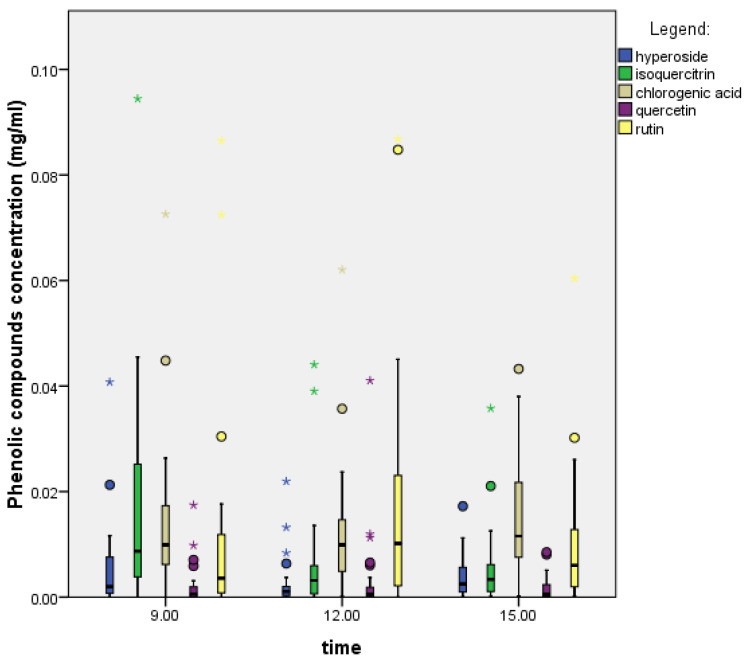
Daily variation in phenolic compounds in the plant species *P. spinosa* (N = 96). The boxplot shows medians (line in each column), quartiles, and standard deviations (vertical lines).

In *P. spinosa* nectar, the ratio of phenolic compounds varied throughout the day (Figure 2). We used the Kruskal–Wallis test, which showed a correlation between the hour of the day and the concentration of (iso)quercitrin (chlorogenic acid: 0.190; rutin: 0.291; (iso)quercitrin: 0.001; hyperoside: 0.067; quercetin: 0.981), and there were statistically significant differences. This means that the time of sampling affected the (iso)quercitrin values. The Mann–Whitney U test showed a statistically significant difference in (iso)quercitrin levels between 9:00 and 12:00 and 9:00 and 15:00.

The concentrations of phenolic compounds (Table 4) were the highest in the morning (total concentration: 0.077 mg/mL) and the lowest in the afternoon (total concentration: 0.036 mg/mL). The levels of rutin and quercetin rose and then fell again, but the opposite happened with hyperoside. Moreover, (iso)quercitrin, which was highest in the morning, decreased the most during the day, while chlorogenic acid increased towards the end of the day.

#### 2.4.2. Crataegus Monogyna

**Figure 3:**
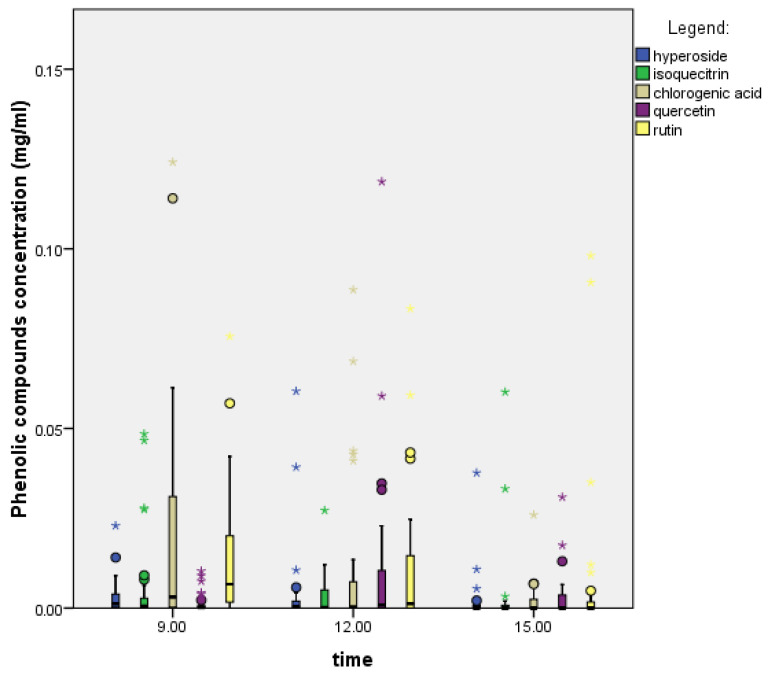
Daily variation in phenolic compounds in the plant species *C. monogyna* (N = 96). The boxplot shows medians (line in each column), quartiles, and standard deviations (vertical lines).

In *C. monogyna* nectar, the ratio of phenolic compounds varied throughout the day (Figure 3). Chlorogenic acid was the most abundant in the morning and afternoon, while rutin was the most abundant at midday. We used the Kruskal–Wallis test, which showed statistically significant differences between the time and the levels of phenolic compounds (chlorogenic acid: 0.000; rutin: 0.000 (iso)quercitrin: 0.001; hyperoside: 0.000; quercetin: 0.031). This means that the time of sampling affects the levels of phenolic compounds. The Mann–Whitney U test showed that there is a statistically significant difference between 9:00 and 15:00 for all phenolic compounds; between 9:00 and 12:00 for chlorogenic acid and rutin; and between 12:00 and 15:00 for all phenolic compounds but chlorogenic acid.

The concentrations of phenolic compounds varied during the day (Table 5). The highest concentrations of phenolics were found at noon (total: 0.087 mg/mL) and the lowest in the afternoon (total value: 0.042 mg/mL). Chlorogenic acid levels fell and then rose again, while the opposite was true for rutin.

## 3. Discussion

Nectar is a major food source for pollinators [2,3,5,6,36,37,38]. Nectar is secreted via nectaries—specialised glands that differ between families [39,40,41,42,43]. Nectar contains water, sugars, amino acids, organic acids, pigments, essential oils, vitamins, minerals, lipid phenols, terpenoids, alkaloids, non-protein amino acids, antioxidants, and other secondary metabolites [3,6,7,9,11,33,44]. Nectar composition is not family-, population-, area-, or individual species-dependent [32,33]. Nectar is influenced by several factors, which can be environmental (abiotic) or biotic. Microclimatic factors and time of the day are considered to have the greatest influence [25,35,45,46,47,48]. Nectar also contains phenolic compounds that either attract pollinators or repel them [8,9].

In our study, we found that the amount of nectar of both species decreased from morning to afternoon. This was most pronounced in *C. monogyna*. The reason for the highest nectar volume in the morning could also be due to accidental (partial) sampling of morning dew by the sampling capillaries. Other studies have found that nectar secretion is the highest between 10:00 and 12:00 [24,25,26,35,49]. However, in our study of phenolic compound concentrations, we found that the concentrations of phenolic compounds were the highest in *P. spinosa* in the morning and in *C. monogyna* at noon, even though the nectar volume was lower at noon compared to the morning. These results may support our assumption that this species secretes very dilute nectar in the morning. Given that bees are mainly more active in the morning and afternoon, the production of more nectar by midday is to be expected [50]. The relative composition of phenolic compounds varies throughout the day, so that, according to our results, the period of the day should influence the ratio of phenolic compounds. To date, no other studies on the effect of time on phenolic compounds have been reported, so our study could serve as a basis for further research.

Phenolic compounds are the most important group of secondary metabolites [51]. Plants produce phenolic compounds mainly for growth, development, and protection. They are particularly important when plants are under biotic or abiotic stress. The amount of phenolic compounds in plants depends on external and internal factors, the age of the plants, climatic influences, and pathogen attack. Increased levels of phenolic compounds can also be a response to UVB radiation, temperature, drought, etc. [52,53]. Some secondary plant metabolites are important for insects because they affect the insect nervous system by binding to neuronal receptor proteins and influencing insect behaviour. They improve memory and help them find food and protect against parasitic diseases [54]. Knowledge of phenolic compounds in nectar is also important from a human point of view, as nectar is the source of honey. Phenolic compounds have medicinal effects on human health [55].

In our study, we focused on five phenolic compounds: rutin, hyperoside, chlorogenic acid, quercetin, and (iso)quercitrin. The most abundant phenolic compound in the nectar of most plant species is thought to be rutin. It is also the most important phenolic compound, as it can protect bees from insecticides [13]. Bees are also attracted to quercetin [16], so in our study we also looked at quercetin and (iso)quercitrin concentrations. Under normal circumstances, queen pheromones inhibit the reproductive potential of workers and the rearing of new queens. However, the study shows that feeding workers with nectar rich in quercetin stimulates their reproductive development (ovary development) and the formation of queen cells, i.e., it promotes queen rearing [15]. Quercetin is also thought to protect bees from pesticides, thereby increasing survival [56]. Two common phenolic compounds in nectar are hyperoside and chlorogenic acid [12,17,18]. Quercetin is also an important phenolic compound for the plants themselves, as it protects plants against biotic and abiotic stresses as an antioxidant [57]. The high proportion of hyperoside could be related to temperature stress in individual plants. It has antioxidant effects and increases cell viability [20]. This is because plants were also sampled at times when it was very hot and UVB radiation was high or the air was cooler than would be expected for that time of year [58].

Rutin is thought to be the most abundant, as studies on various plant species within the Rosaceae family have also shown [59,60]. Chlorogenic acid is often the most abundant phenolic acid in nectar [61,62], but there are no known data for this family. Interestingly, the genus *Crataegus* is considered a nectar source rich in hyperoside [20], yet in our nectar analysis, it was the least abundant of all the phenolic compounds examined. It is also interesting that we observed a large number of bees despite the nectar containing high levels of chlorogenic acid, which is considered a repellent for bees [63]. Our results showed that *C. monogyna* contained the highest concentration of chlorogenic acid, which is also considered a common phenolic compound in nectar [12,17,18]. It is considered to be toxic to bees, and high levels may deter bees [8,19] or protect plants from pathogen infection [64]. The higher amount of chlorogenic acid in *C. monogyna* in our study could be due to the plant’s response to pollinator stress. It should be noted, however, that such small amounts of chlorogenic acid are not toxic to bees [8,63]. However, given that quercetin is supposed to be one of the most abundant flavonoids in plants [14], it was surprising finding that its overall levels were very low.

According to our results, the phenolic compounds (rutin and (iso)quercitrin) in *P. spinosa* are influenced by air and soil temperature and soil moisture. When soil and air temperatures rise, the concentration of rutin in *P. spinosa* nectar also increases. Higher temperatures are thought to increase the breakdown of rutin. Kadakal and Duman [65] conducted their research at very high temperatures, which are not even possible in nature, except in the case of fire. UVB radiation and absolute humidity have no effect on the concentration of phenolic compounds in *P. spinosa* nectar.

In *C. monogyna*, temperature also has an effect, but the rutin concentration decreases. In this plant species, soil and air temperatures influence almost all the phenolic compounds tested, except for quercetin. Higher soil and air temperatures lead to a reduction in hyperoside concentrations. Higher soil and air temperatures and higher UVB radiation lead to a reduction in (iso)quercitrin concentrations. However, almost all abiotic factors, except soil moisture, lead to lower concentrations of chlorogenic acid and rutin.

To generalise, we can say that only air and soil temperatures influence the concentration of some phenolic compounds. But we can surely assume that all investigated factors affect nectar quantity. There is a lack of research on the influence of abiotic factors on phenolic compounds in nectar, so it would be good to do more in the future to learn more about phenolic compounds in nectar and what influences them.

## 4. Materials and Methods

### 4.1. Selected Species

This study included 2 plant species of the Rosaceae family. The species were selected based on seasonal dynamics within the family [21,66] and the size of their populations in the wild in Slovenia [66,67]. Both species are native, and they were also selected based on previous surveys of these species combined with observations of pollinator pasture.

**Figure 4:**
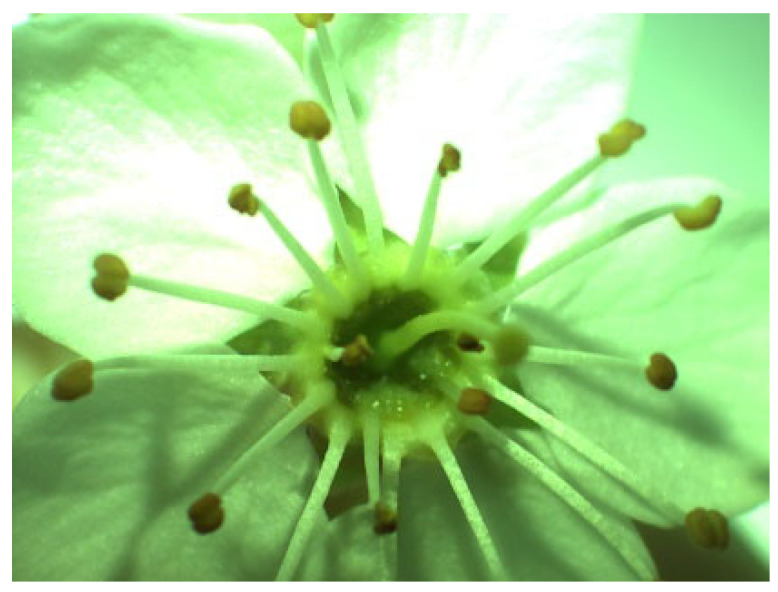
Flower *P. spinosa* under a microscope loupe (magnification 10×).

*P. spinosa* is a shrub species found in woodlands, hedgerows, and rocky slopes. The branches are strongly thorny, and flowering occurs before leaking out. It flowers between March and May and has white, wreath-like flowers [66]. Nectar is secreted in base of the hypanthium (Figure 4).

**Figure 5:**
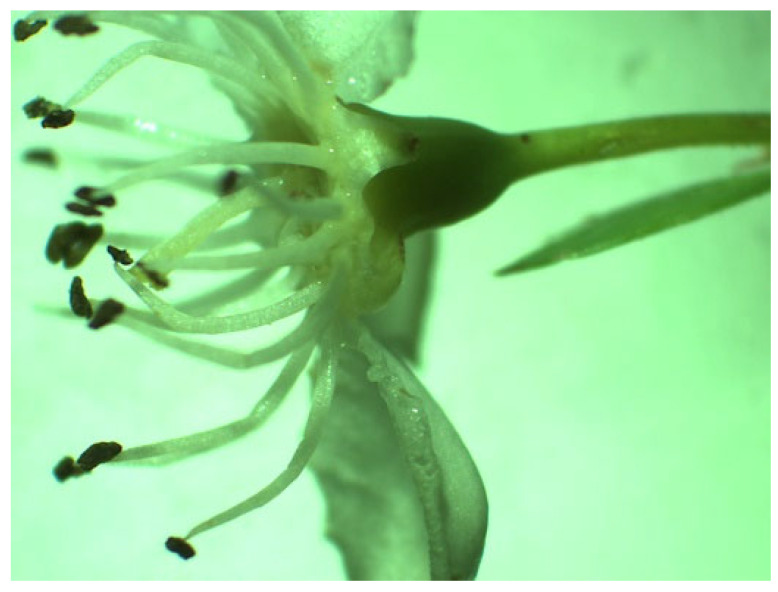
The flower *C. monogyna* under a microscope loupe (magnification 10×).

*C. monogyna* is also a shrubby plant species, growing on rocky grassy slopes, hedges, and woodland slopes and in light woodlands. The branches are thorny. It has small white flowers and blooms from May to June. The sepals are much smaller than the petals. The flowers are in inflorescences [66]. Nectar is secreted in base of the hypanthium (Figure 5).

### 4.2. Sampling Locations

**Figure 6:**
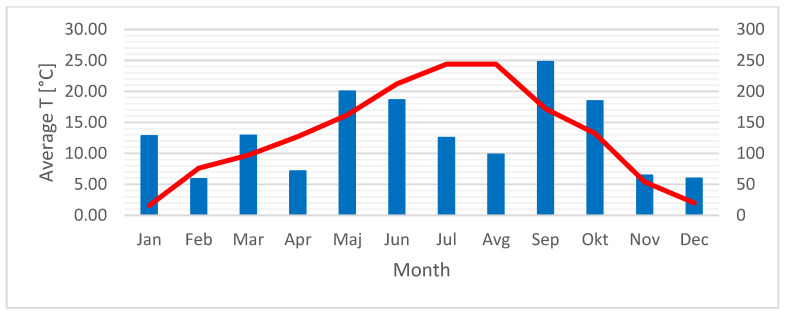
Average annual temperature (red) and precipitation (blue) in 2024 (ARSO, 2025).

The survey took place in Slovenia, in the capital Ljubljana. Slovenia is located at the crossroads of four biogeographical regions: the alpine, Pannonian, Dinaric, and Sub-Mediterranean regions. It lies in a warm temperate zone. Due to its transitional location, it is influenced by several climates: alpine, continental, and Mediterranean [68]. Each plant species was sampled at two different locations in the wild. Both species were sampled over a period of one year. We sampled half of the samples in one location and half in the other. The average annual temperature and precipitation in 2024 are shown in Figure 6.

**Figure 7:**
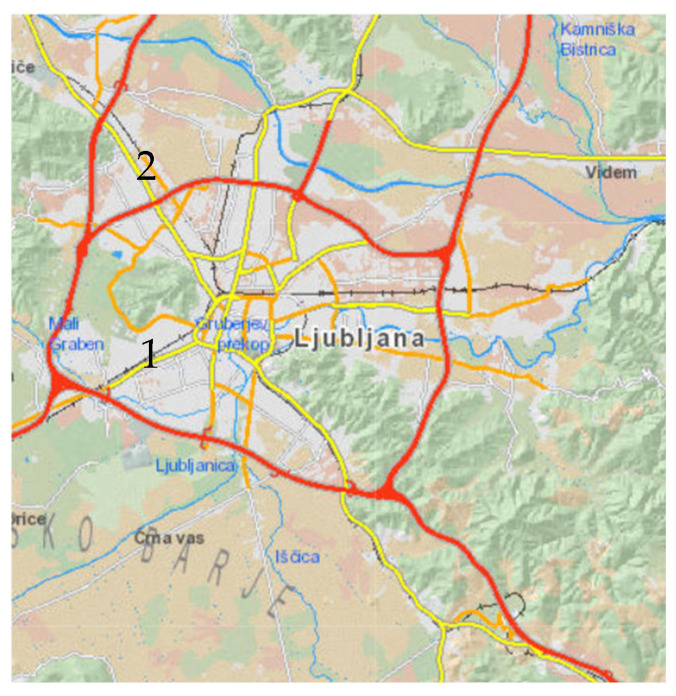
Map of surveyed areas in Ljubljana and its surroundings. The locations are marked with numbers (1—Biological Centre on Večna pot; 2—Roje at Šentvid).

Location 1: Večna pot Biological Centre in Ljubljana (Figure 7)

This is a wet meadow site with typical marsh plants at 295 metres above sea level. Along the edge there are woods and several communities of different types of shrubs. The ground here is often flooded, even in the forest area, especially in spring.

Location 2: Meadow in Roje (Figure 7)

The meadow with the forest edge is located 298 metres above sea level. It is a dry meadow with sections of shrubby communities [69]. The soil is limestone with deposits from the nearby Sava River. The edges of the meadow are woodland and arable land. The meadow is mown once a year, while the edge of the forest is not maintained and becomes heavily overgrown and spreads into the meadow.

### 4.3. Nectar Sampling

Because pollinators visit the flowers regularly, it is important to protect the flowers beforehand to prevent pollinators from sipping nectar. Various veils, gauze, nets, etc., can be used as protection [28,32,70]. The flowers of the specimens were protected with a veil one day before sampling. We included 10 flowers per sample. This means that we did not cover the whole plant, but only the parts of the specimen that were studied. As nectar production and persistence are also affected by rain [46], we sampled when there was no rain.

The nectar was sampled three different times during the day: at 9:00, 12:00, and 15:00. The sampling always took place on the same day at the same location. Flowers were sampled using microcapillary tubes. One-microliter microcapillaries from Vitrex Medical were used for sampling. For each plant species, four samples were taken at each selected time during field sampling. Microcapillary sampling works by capillary suction. Latex gloves were used to handle them. After sampling, the liquid level in the capillary was measured to calculate the sampled nectar volume (the latter being proportional to the liquid level). The microcapillary tubes with the collected nectar were placed in centrifuge tubes and stored in a −20 °C freezer, where they remained until further analysis. A total of 96 samples were collected per plant species.

We also measured abiotic factors for each location, date, and time. We measured air and soil temperature, air and soil humidity, and UVB. Soil parameters were measured at a depth of 7 cm (the length of the sensors of the measuring instrument) and air parameters were measured at the height of the flowers of the sampled species.

Samples from the freezer were thawed, transferred to vials for further analysis, and diluted with 150 µL of distilled water. The centrifuge tubes were placed on a centrifuge three times, for 1 min each (at 12,000× *g*). After the final centrifugation (3 min), the liquid was transferred to an HPLC vial. We adapted an existing HPLC method for phenolics and prolonged the flush times in order to hinder sugar precipitation in the column. Otherwise, the high sugar concentration did not interfere with the separation of phenolics.

### 4.4. Analysis of Phenolic Compounds

Standards for phenolic analysis (chlorogenic acid, rutin, quercetin, quercitrin, (iso)quercitrin, and hyperoside, Extrasynthese, Genay, France) were prepared by dissolving 5 mg of each standard in 10 mL of methanol (Merck, Darmstadt, Germany). The stocks were then combined and diluted with 50% (*v*/*v*) methanol to produce a working standard solution of 20 µg/mL for each analysis. Properly diluted nectar samples were analysed using a Vanquish UHPLC system (Thermo Scientific, San Jose, CA, USA) coupled to a UV detector (detection wavelengths set to 330 nm and 360 nm) and Chromeleon 7.2 SR4 data acquisition software (Thermo Scientific). The separation column was a Hypersil Gold C18 column with dimensions of 100 mm × 2.1 mm i.d. and a particle size of 3 µm (Thermo Scientific) at 40 °C. The solvents for the gradient elution were water with 0.1% formic acid (solvent A) and acetonitrile with 0.1% formic acid (solvent B). The elution programme consisted of an isocratic elution from 0 to 7 min in 10% solvent B, followed by a gradient elution from 7 to 14 min in 10% to 60% solvent B. The column conditioning consisted of an isocratic elution from 14.1 min to 19 min at 10% solvent B. The flow rate was kept constant at 0.25 mL/min. The analysis time was 19 min. The sample vials were regulated with a thermostat at 10 °C. The autosampler flushing liquid was water with 10% methanol (*v*/*v*). The injection volumes were 5 µL for standard solutions and 10 µL for samples. For more information about the descriptive statistics of the phenolic compound analysis method see Table 6.

### 4.5. Data Processing

The plotted HPLC chromatographs were first analysed and then the concentrations were calculated from the standards. The calculated amounts were normalised by calculating the amounts per single flower.

The basic data processing was carried out in Excel. For the effect of abiotic factors on nectar, we used SPPS and calculated Spearman correlation coefficients. We first checked our data using the Kolmogorov–Smirnov test, which showed that the data were not normally distributed, so we then chose the Spearman correlation coefficient to show which abiotic factors influence which compound or quantity.

For the effect of time on the compounds, we also first applied the Kolmogorov–Smirnov test and found that the data were abnormally distributed. To determine whether there are statistical differences between the sampling time and compound concentrations, we first used the Kruskal–Wallis test. If we found statistical differences with this test, we then used the Mann–Whitney U test to see which exact hours and compounds there were statistical differences between, again in IBM SPSS. Statistics 23.

The graphs of the effect of the time on the compounds were produced in SPPS and the others in Excel.

## 5. Conclusions

*P. spinosa* and *C. monogyna* are common woody plant species in Slovenia and are considered important melliferous plants. We investigated phenolic compounds that play an important role in bee biology and in the interaction between bees and plants (including rutin, quercetin, isoquercitrin, hyperoside, and chlorogenic acid). We wanted to know how rich in phenolic compound concentrations these two species are as they flower at different times and if they are both good sources of bee forage. The flowers secrete nectar throughout the day, and each plant has many flowers. The nectar of both plant species is dominated by sugars, glucose, and fructose, with some variation in the phenolic compounds. Among the phenolic compounds investigated, rutin is the most abundant in *P. spinosa* nectar, while chlorogenic acid is the most abundant in *C. monogyna* nectar. Both types of nectar contain hyperoside as the smallest component. The time of day influences the concentration of phenolic compounds in the nectar; the concentration of phenolic compounds is higher at midday in *C. monogyna*, whereas in *P. spinosa*, the concentration of phenolic compounds is higher in the morning, as is nectar secretion. The scattered data on the effect of abiotic factors on the nectar of both plant species makes it difficult to confirm whether they affect the concentration of phenolic compounds. It would be interesting to know more about the relationship between the concentration of phenolic compounds and bee behaviour. Additional observations and investigations are needed to answer this question in the future.

## Figures and Tables

**Table 1:** Average concentration of the phenolic compounds studied in the plant species studied (N = 96 for each species). Values are also given as median ± standard deviation (SD).

	chl. Acid (μg/mL)	Rutin (μg/mL)	(iso)quercitrin (μg/mL)	Hyperoside (μg/mL)	Quercetin (μg/mL)
*P. spinosa* (mean)	14.6	17.2	13.8	6.1	2.22
*P. spinosa* (median ± SD)	10.3 ± 17.2	6 ± 33.6	4.7 ± 46.5	1.8 ± 23.2	0.6 ± 5
*C. monogyna* (mean)	22.0	11.0	4.0	3.0	21.0
*C. monogyna* (median ± SD)	0.5 ± 86.7	1.4 ± 20.4	0.19 ± 10.6	0.4 ± 8.6	0.09 ± 136

**Table 2:** Spearman’s correlation coefficient of abiotic factors in *Prunus spinosa* nectar. Only statistically significant correlations are shown. x means there is no statistically significant correlation.

	Air T (°C)	Soil T (°C)	Soil Humidity (%)	Absolute Air Humidity (g/kg)	UVB (μW/cm^2^)
chloro. acid	x	x	x	x	x
rutin	0.257 *p* = 0.012	0.201 *p* = 0.049	0.244 *p* = 0.017	x	x
(iso)quercitrin	x	−0.315 *p* = 0.002	x	x	x
hyperoside	x	x	x	x	x
quercetin	x	x	x	x	x

**Table 3:** Spearman’s correlation coefficient of abiotic factors for *Crataegus monogyna* nectar. Only statistically significant correlations are shown. x means there is not a statistically significant correlation.

	Air T (°C)	Soil T (°C)	Soil Humidity (%)	Absolute Air Humidity (g/kg)	UVB (μW/cm^2^)
chloro. acid	−0.425 *p* = 0.000	−0356 *p* = 0.000	x	−0.236 *p* = 0.021	−0.376 *p* = 0.000
rutin	−0.366 *p* = 0.000	−0.340 *p* = 0.001	x	−0.202 *p* = 0.048	−0.279 *p* = 0.006
(iso)quercitrin	−0.326 *p* = 0.001	−0.321 *p* = 0.001	x	x	−0.260 *p* = 0.011
hyperoside	−0.290 *p* = 0.004	−0.309 *p* = 0.002	x	x	x
quercetin	x	x	x	x	x

**Table 4:** Average concentrations of phenolic compounds during the day in the plant species *P. spinosa*. Values are also given as median ± standard deviation (SD).

	chl. Acid (μg/mL)	Rutin (μg/mL)	(iso)quercitrin (μg/mL)	Hyperoside (μg/mL)	Quercetin (μg/mL)
09:00 (mean)	17	16	30	12	22
09:00 (median ± SD)	9.9 ± 25.1	4.4 ± 38	8.7 ± 77.9	1.9 ± 39.5	0.6 ± 3.5
12:00 (mean)	12	25	6	3	3
12:00 (median ± SD)	9.9 ± 11.7	10 ± 41.5	3.1 ± 9.9	1.7 ± 4.5	0.6 ± 7.5
15:00 (mean)	15	10	5	4	2
15:00 (median ± SD)	11.6 ± 10.9	6 ± 12.2	3.4 ± 7.1	2.5 ± 4.1	0.6 ± 2.2

**Table 5:** Average concentrations of phenolic compounds during the day in the plant species *C. monogyna.* Values are also given as median ± standard deviation (SD).

	chl. Acid (μg/mL)	Rutin (μg/mL)	(iso)quercitrin (μg/mL)	Hyperoside (μg/mL)	Quercetin (μg/mL)
09:00	30	14	6	3	1
	3 ± 60.5	6 ± 17.9	0.6 ± 12.8	12 ± 4.8	0.1 ±2
12:00	11	11	3	4	58
	0.3 ± 21.9	1.2 ± 19.8	0.2 ± 5.8	0.3 ± 12.4	0.8 ± 233.2
15:00	26	8	3	2	3
	0 ± 135	0 ± 23.5	0 ± 11.9	0 ± 6.8	0 ± 6.5

**Table 6:** Descriptive statistics of the phenolic compound analysis method.

Analyte	Repeatability (% RSD; *n* = 5)	Reproducibility (% RSD; *n* = 3)	LOD (ng) *	LOQ (ng) *	Linearity Range (ng *; r)
Proline	2.82	5.37	0.7	2.3	2.3–200; 0.9993
Leucine/Isoleucine	2.07	3.71	0.9	3.0	3.0–200; 0.9995
Methionine	4.74	6.39	1.9	6.4	6.4–200; 0.9988
Alanine	0.58	4.38	0.1	0.5	0.5–200; 0.9958
Tyrosine	1.96	2.53	0.2	0.5	0.5–200; 0.9992

* The values refer to the analyte amount injected into the column; LOD—limit of detection; LOQ—limit of quantification.

## Data Availability

The original data presented in the study are openly available in the repository of University Botanic Gardens Ljubljana, Biotechnical faculty.
